# Artificial Intelligence Algorithms for Benign vs. Malignant Dermoscopic Skin Lesion Image Classification

**DOI:** 10.3390/bioengineering10111322

**Published:** 2023-11-16

**Authors:** Francesca Brutti, Federica La Rosa, Linda Lazzeri, Chiara Benvenuti, Giovanni Bagnoni, Daniela Massi, Marco Laurino

**Affiliations:** 1 Institute of Clinical Physiology, National Research Council, 56124 Pisa, Italy; fbrutti@ifc.cnr.it (F.B.); larosa.fed@gmail.com (F.L.R.); chiara.benvenuti@ifc.cnr.it (C.B.); 2 Uniti of Dermatologia, Specialist Surgery Area, Department of General Surgery, Livorno Hospital, Azienda Usl Toscana Nord Ovest, 57124 Livorno, Italy; lazzeri.linda@gmail.com (L.L.); giovanni.bagnoni@uslnordovest.toscana.it (G.B.); 3 Department of Health Sciences, Section of Pathological Anatomy, University of Florence, 50139 Florence, Italy; daniela.massi@unifi.it

**Keywords:** melanoma, Artificial Intelligence, dermoscopic images, machine learning, deep learning

## Abstract

In recent decades, the incidence of melanoma has grown rapidly. Hence, early diagnosis is crucial to improving clinical outcomes. Here, we propose and compare a classical image analysis-based machine learning method with a deep learning one to automatically classify benign vs. malignant dermoscopic skin lesion images. The same dataset of 25,122 publicly available dermoscopic images was used to train both models, while a disjointed test set of 200 images was used for the evaluation phase. The training dataset was randomly divided into 10 datasets of 19,932 images to obtain an equal distribution between the two classes. By testing both models on the disjoint set, the deep learning-based method returned accuracy of 85.4 ± 3.2% and specificity of 75.5 ± 7.6%, while the machine learning one showed accuracy and specificity of 73.8 ± 1.1% and 44.5 ± 4.7%, respectively. Although both approaches performed well in the validation phase, the convolutional neural network outperformed the ensemble boosted tree classifier on the disjoint test set, showing better generalization ability. The integration of new melanoma detection algorithms with digital dermoscopic devices could enable a faster screening of the population, improve patient management, and achieve better survival rates.

## 1. Introduction

In recent decades, the incidence rates and the number of diagnoses of cutaneous melanoma have constantly increased both at global and Italian levels [1]; in 2020, about 14,900 new skin melanoma diagnoses were estimated in Italy (male = 8100, female = 6700), and the trend of incidence appears to increase significantly both in males (+4.4% per year) and in females (+3.1% per year) [2].

The risk of skin melanoma is linked to genetic, phenotypic, and environmental factors and combinations among them [3]. Early identification and surgical excision of malignant lesion are the most important interventions in preventing metastatic disease and decreasing mortality [4]. In the standard diagnostic process, the first step, performed by dermatologist specialists, consists in the naked-eye evaluation of the skin lesion using the ABCDE criteria. The evaluation includes the assessment of the asymmetry of the lesion, the irregularity of the border, color variegation, diameter greater than 6 mm, and evolution. These criteria have been developed to help general doctors not specialized in dermatology to make the first screening for differentiating benign and malignant lesions [4]. The current gold standard for the diagnosis of a skin lesion is the use of a dermatoscopy tool. This allows dermatologists to measure the above criteria in an accurate and fast way. Moreover, it is widely available and easy to use [5]. This non-invasive technique aids in the differentiation between benign and malignant skin lesions, mainly on the basis of the color and the structure of pigmentation. The color indicates the depth of the lesion in the dermis, while the structure reflects the anatomy of the lesion [6]. However, dermoscopic features differ considerably among lesions at different sites, and lesions at certain sites may show a particular and unique appearance, so extensive training is needed for both dermatologists and general practitioners to avoid the wrong classification of lesions [6]. One of the most important technological perspectives in the use of digital dermoscopy for the diagnosis of skin cancer is automatic analysis performed by Artificial Intelligence (AI) algorithms [7]. Esteva et al. [7] investigated the effectiveness of AI-based solutions, comparing their results with the diagnosis of expert dermatologists regarding three diagnostic tasks. In a similar way, Pham et al. [8] proposed an AI model for melanoma detection and compared it with 157 dermatologists. Both works achieved an accuracy score consistent with those of the experts, demonstrating the possibility of using AI tools in clinical practice. Hence, an automatic procedure able to analyze skin lesions and detect the malignant ones would be useful to assist dermatologists in diagnosis and overcome inter-observer variability. Recently, several studies have proposed useful solutions to classify dermoscopic images in benign vs. malignant skin lesions through the use of machine learning (ML) [9,10] and deep learning (DL) techniques [8,9,10,11,12,13,14].

DL is a subset of ML that uses neural networks with multiple internal layers to analyze data. The main difference between ML and DL lies in the requirement of initial structured data: ML needs a priori categorized data and thus a separate feature extraction phase, while DL automatically extracts features and attributes from a dataset for training. Therefore, ML techniques require user intervention to structure data, extract features, and correct errors, while DL requires little or no user intervention.

Some of the problems to be addressed in the classification of skin lesions are the variability in image samples and the imbalance in the number of samples per class in open-access datasets, usually in favor of benign skin lesions. Most of the studies mentioned above implemented models by training and testing them on images belonging to the same dataset, avoiding evaluating the problem of image variability and the generalization capability of the developed AI solutions. In fact, using the same dataset for both training and testing implies that the test set shares the same characteristics as the training set and may cause a bias in accuracy calculation. In addition, the problem of class imbalance is usually addressed by performing massive data augmentation on malignant samples in order to increase the number of malignant samples with respect to that of benign ones.

In our study, we propose two different AI models for the skin lesion dermoscopic image classification task: a traditional ML-based method and a DL-based one. We compare the performance of the two approaches to evaluate the best AI method and measure it against the current state of the art. We use three different datasets for the training step and a disjoint distinct dataset to test the accuracy of the two different approaches. Moreover, to ensure a balanced dataset, we create 10 different, randomly selected balanced datasets from the training dataset and consider the average of the performance for the final evaluation.

## 2. Materials and Methods

**Figure 1:**
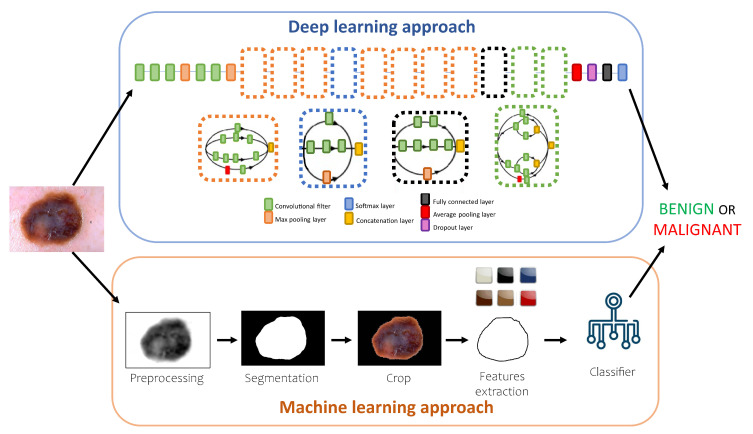
Pipeline followed to classify a dermoscopic image using two different approaches. The DL approach shows the neural network architecture used in this work.

Figure 1 describes the pipeline of the two AI approaches for dermoscopic image classification used in this work. On one hand, a DL-based method was investigated through the use of a pre-trained CNN with a specific architecture. On the other hand, an ML-based approach involving a segmentation algorithm and the extraction of features was explored. The comparison between the performance of the different methods in terms of accuracy and computational cost allowed us to determine the best approach.

### 2.1. Dataset

**Figure 2:**
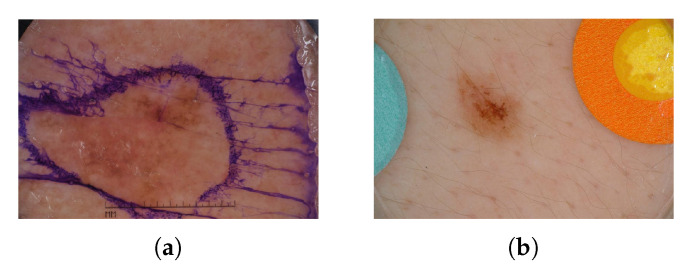
Examples of deleted images from the ISIC 2019 dataset. Pen marks (**a**). Colored patches (**b**).

In this study, three different open datasets collected from online available datasets were used to develop and test the two classification approaches (Table 1). Part of the used dermoscopic images came from the International Skin Imaging Collaboration (ISIC) 2019 challenge [15,16,17] dataset (DB1); another part of the dataset came from an open dataset (DB2) available upon request [18]; and the last part of the dataset was provided by Pedro Hispano Hospital (PH2) [19] (DB3). From DB1, we deleted images containing pen marks and colored patches to avoid any possible confounding factor (Figure 2). Since the malignant skin lesions in DB1 are significantly fewer than the benign ones (9281 vs. 14,830), we performed 10 random dataset shuffling operations by extracting, each time, only 65% of the entire DB1 benign dataset. In this way, we obtained ten different balanced datasets with the same number of images equally distributed between benign and malignant diagnoses. Both machine learning and deep learning models were trained on the same ten datasets to compare the distributions of the classification performance of the two models considering the same inputs.

### 2.2. Machine Learning Approach

As described in Figure 1, the traditional ML approach consists of a preprocessing step to prepare the dermoscopic image for the segmentation and crop steps, and a crop feature extraction procedure to collect the parameters for the classification step.

#### 2.2.1. Preprocessing and Segmentation

Since the used datasets do not provide ground-truth segmentation of the skin lesions, a segmentation algorithm was adopted. Following the method by Vandaele et al. [20], persistent homology was implemented to artificially destruct irrelevant objects and separate the relevant region from the background noise. The method [20] uses the Ripser python library [21] to compute Vietoris–Rips persistence diagrams representing the birth and the death of the image components. Then, once a threshold is selected, the method considers the objects with a lifespan greater than the threshold and increases the contrast between them and the background. Since two connected components have a significantly longer lifespan than all others, the algorithm allows for the extraction of only the connected components of the image.

Once each image was preprocessed, an isocontour algorithm, based on identifying iso-valued contours in the image, was applied to obtain skin lesion segmentation. Isocontour was adopted in agreement with Vandaele et al. [20]. The preprocessing and segmentation of the dermoscopic images were performed by coupling the open-source software library OpenCV [22] and the lean persistent homology package “Ripser.py” [21] using Python 3.7 on a Windows operating system.

#### 2.2.2. Feature Extraction and Classification

**Figure 3:**
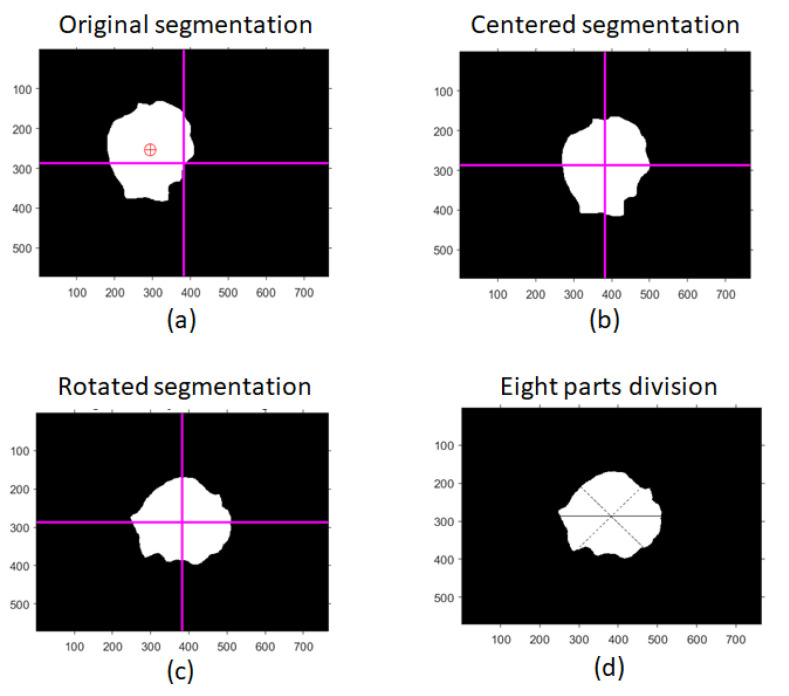
First steps in feature extraction and classification starting from skin lesion segmentation. The original segmentation image (**a**) was centered (**b**) and rotated along the longer axis (**c**). Finally, the segmentation image was divided into eight parts (**d**).

Once the segmentation result was obtained, feature extraction was carried out. First, both image and segmentation image were recentered and rotated along the longer axis in order to have a standardized reference system. Then, both images were divided into eight parts to assess the shape and color asymmetry of the skin lesion (see Figure 3). Indeed, according to the ABCDE rule [23] and following the parameters extracted by Bhuiyan et al. [24], the following image features were collected (see Table 2):

- Shape asymmetry.
- Border irregularity.
- Fractal dimension index.
- Compactness index.
- Color density.
- Color asymmetry index.
- Standard deviation (SD) of the color distribution.

The work by Dalila et al. [25] showed that most of the relevant features for skin lesion classification are related to color variation; hence, we paid more attention to that type of features. To extract the colors from the images, the K-means algorithm was applied. We obtained the three dominant colors of the skin lesion from the segmented image, and the minimum Euclidean distance was computed to associate each detected color to one of the a priori registered colors. Then, we assessed the color asymmetry index by extracting the frequency of each gray-level value on each of the 8 image regions and computing the color histogram difference between two opposite regions.

To assess shape asymmetry, the Normalized E-Factor (NEF) [26] was computed. It has been shown to be useful for shape description, to measure digital compactness with or without holes, and to overcome some drawbacks that are present in the classical and normalized discrete compactness measures. Once the features were collected, different types of ML classifiers were trained to classify benign and malignant skin lesions. Among KNN, SVM, and decision tree classifiers, ensemble boosted tree performed the best and was selected as the ML model. Analysis and classification were performed by using MATLAB software (R2022a; Natick, MA, USA; The MathWorks Inc.).

**Table 2:** Summary of the extracted features for the training of the machine learning-based model. The output range was obtained through the normalization of the output values of each feature.

Extracted Feature	Formula/Algorithm	Source	Output Range
Shape asymmetry	NEF	[23,26]	[0–5]
Border irregularity	$\frac{4\pi A}{P^{2}}$	[11]	[0–5]
Fractal dimension index	Box-counting algorithm	[24]	[0–5]
Compactness index	$\frac{P^{2}}{4\pi A}$	[27]	[0–5]
Color density	K-means +	[11]	[0–100]%
Color asymmetry index	Minimum Euclidean distance	[23,26]	[0–5]
Standard deviation (SD) of the color distribution	(White, black, red, light brown, dark brown, blue-gray)	[11]	[0–100]

### 2.3. Deep Learning Approach

The DL approach was performed by using a CNN model based on a pre-trained (on the ImageNet database [28]) Inception-v3 model trained with the hyperparameters reported in Table 3. The Inception-v3 architecture consists of 11 inception modules, where each module consists of pooling layers and convolutional filters, with rectified linear units as the activation function, as it can be seen in Figure 1. To adapt the images of the datasets to the input size of the CNN, each image was resized to 299 × 299 × 3. The dataset was divided into three sets: DB1 and DB2 were merged and used as training (70%, 13,952 images) and validation sets (30%, 5980 images) to train and tune the model in skin lesion classification, while DB3 was used as a disjoint test set to independently assess model performance. Data augmentation was performed using random rotations, shifts, and zooms on the training and validation sets, using the parameters in Table 3. Training was performed using MATLAB software (R2022a; Natick, MA, USA; The MathWorks Inc.) and Deep Learning Toolbox. To evaluate the model performance in skin lesion classification, accuracy, specificity, sensitivity, and precision metrics were computed [29].

## 3. Results

In the following paragraphs, we will discuss the results obtained with our models. First, we will present how we selected the dataset after various image analyses; then, we will report the metrics of the two approaches in the validation and test phases.

The metrics used for ML and DL performance evaluation were computed as follows:

$$\begin{array}{cc} \; & Accuracy=\frac{TP+TN}{TP+TN+FP+FN}\\ \; & Specificity=\frac{TN}{TN+FP}\\ \; & Sensitivity=\frac{TP}{TP+FN}\\ \; & Precision=\frac{TP}{TP+FP} \end{array}$$

where TP, TN, FP, and FN refer to the numbers of true positives, true negatives, false positives, and false negatives, respectively.

### 3.1. Dataset

From DB1, 94 and 15 images of benign and malignant skin lesions, respectively, were deleted because of the presence of pen marks (Figure 2a). Similarly, 111 benign images were deleted from DB1 due to the presence of colored patches (Figure 2b). Hence, the final combined dataset (DB1 and DB2) for the training and validation steps was formed by 25,122 skin lesion images. From the combined dataset, we extracted ten subsets balanced in the numbers of malignant and benign images. Each of the ten equally distributed datasets included 19,932 images.

### 3.2. Machine Learning Approach

**Figure 4:**
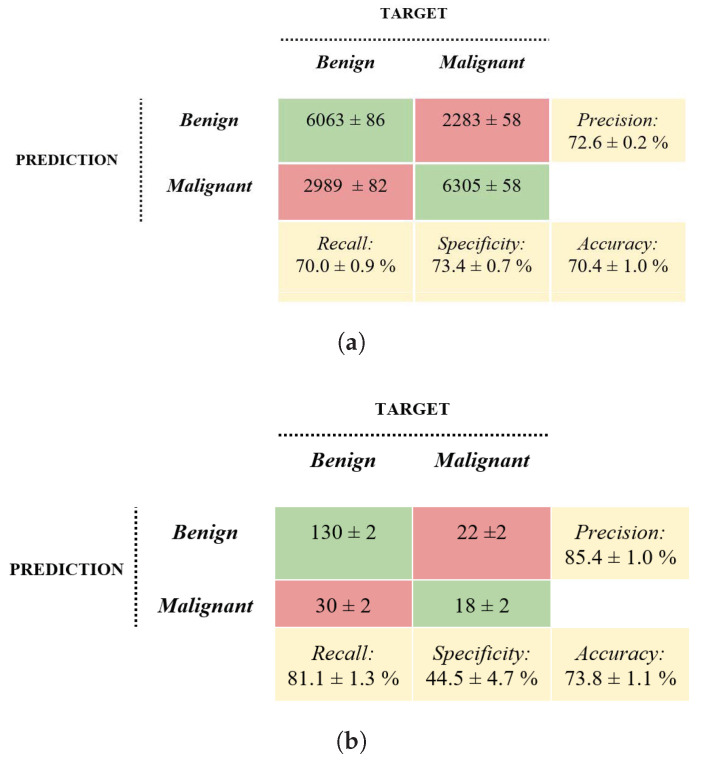
Confusion matrices. Machine learning approach’s average results on the validation set (**a**) and the DB3 disjoint test set (**b**).

The average accuracy, sensitivity, specificity, and precision of the ML approach in the test phase were 73.8%, 81.1%, 44.5%, and 85.4%, respectively. The averages and the standard deviations of the performance metrics of the ten machine learning training operations on the validation set and the DB3 disjoint test dataset are reported in the confusion matrices in Figure 4.

**Figure 5:**
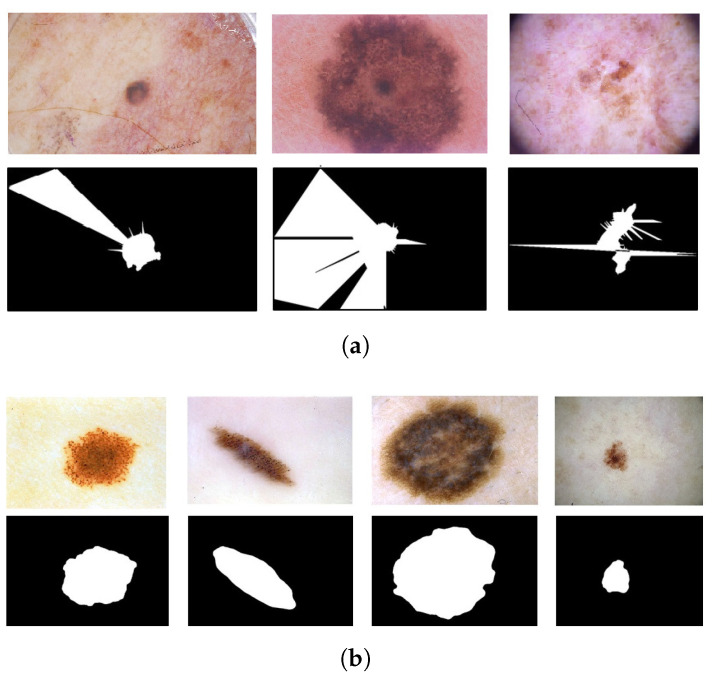
Examples of failed (**a**) and correct (**b**) segmentation results with the correspondent original images.

In addition, we evaluated the error rate of the preprocessing and segmentation algorithm, which failed on a few images of the training dataset. After manual visual evaluation, we determined the error rates of 7.5% and 6.1% for malignant and benign images, respectively (Figure 5).

### 3.3. Deep Learning Approach

**Figure 6:**
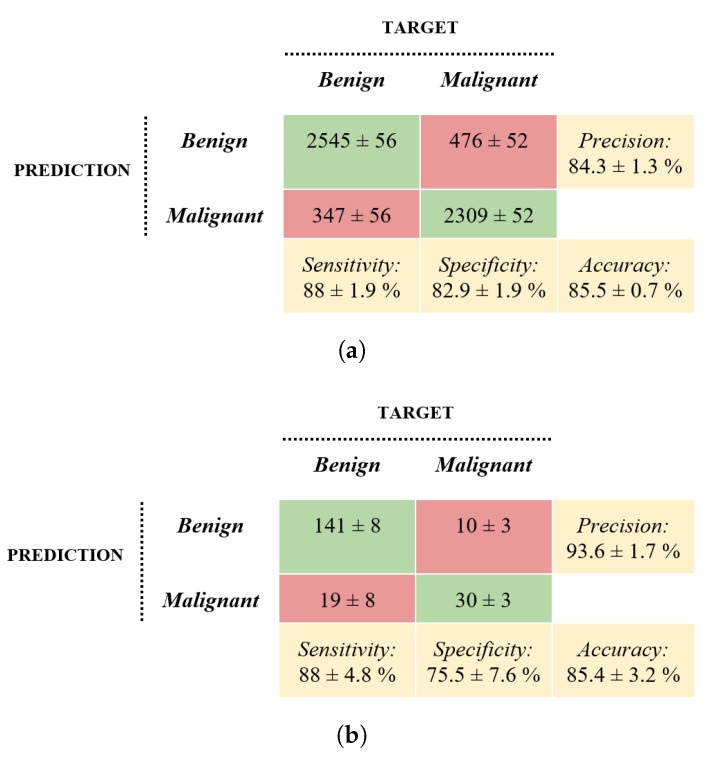
Confusion matrices. Deep learning approach’s average results on the validation set (**a**) and the DB3 disjoint test set (**b**).

Figure 6 shows the confusion matrices of the averaged performance metrics of the ten trained CNN-based models. The evaluation was made on both validation and disjoint DB3 datasets. The average accuracy, sensitivity, specificity, and precision of the DL approach in the test phase were 85.4%, 88%, 75.5%, and 93.6%, respectively.

## 4. Related Works and Discussion

This work aims to investigate the best AI-based approach for dermoscopic skin lesion image classification into benign and malignant lesions. We developed two different ML and DL models using images from different open datasets with large variability. We tested both AI approaches on a disjoint dataset to evaluate their effective generalization capabilities.

With our work, we would like to underline the importance of using completely disjoint and independent datasets between training and test activities in ML approaches in order to correctly evaluate the effective performance, robustness, and generalization capabilities of the trained ML and DL models in possible real applications. In fact, the use of separate databases between the training and test phases is not such a common practice, given that this often leads a reduction in the classification performance metrics (such as accuracy, specificity, sensitivity, and precision) on the test datasets used. However, it is necessary to point out the practice of separating datasets in order to be able to correctly evaluate the real capabilities of the developed ML and DL models, both in terms of classification performance and generalization capability, in real application contexts.

In our work, the average accuracy of 85.4% achieved by the DL model on the disjoint test set outperformed the ML model (average accuracy of 73.8%) and also the current state-of-the-art classifiers, as discussed in the following. Table 4 summarizes the main results of literature classification approaches for dermoscopic skin lesion analysis, considering only the ones that provide classification between benign and malignant skin lesions. Other works [8,30,31,32,33,34] investigated different classification aims (disease-based, common nevus vs. melanoma, etc.) with respect to our work. In particular, López-Labraca et al. [32], Xie et al. [33], and He et al. [34] achieved good performance in the global accuracy of malignant vs. all and seborrheic keratosis vs. all classification on a disjoint dataset. Their training dataset was formed by common nevi, melanoma, and seborrheic keratosis images and contained the ISIC 2017 dataset [17].

As in our study, the datasets of the works reported in Table 4 were obtained from different available open datasets. However, in all these studies, the training and test images belonged to the same dataset. Premaladha and Ravichandran [12] used the Contrast Limited Adaptive Histogram Equalization technique (CLAHE) to preprocess dermoscopic skin lesion images and obtain a contrasted image to accurately derive the features. DL-based neural networks (DLNNs) and Hybrid AdaBoost algorithms were used for the classification of skin lesions (accuracy of 90%). Khan et al. [11] developed five different models based on five different databases. They split each database into two equal parts: one was used for training and one for testing. They used Support Vector Machine (SVM) for classification (accuracy of 98.4%). Mahbod et al. [14] and Liu et al. [10] proposed a transfer learning-based model by extracting features from pre-trained CNNs. The first one used the extracted features directly as input of an SVM classifier (accuracy of 91.4%), while the second one obtained mid-level feature representations by utilizing the relationships among different image samples based on distance metric learning (accuracy of 92.1%).

Among the listed methods, the performance of our model is consistent with the one reported in the work by Bechelli and Delhommelle [9] (accuracy of 73% for ML and 88% for DL); but, unlike them, our number of images is larger, and our model performance evaluation is computed on a disjoint test dataset. Testing methods on a disjoint set of images enables one to assess the real accuracy of a model, since dermoscopic image variability is quite large between training and test sets.

To the best of our knowledge, only the work by Kaur et al. [13] proposed a disjoint dataset for the testing of benign vs. malignant skin lesion image classification. They developed a novel DCNN, and they trained the model on the online ISIC database (ISIC 2016 [36], 2017 [17], and 2020 [37]). They tested their model on these datasets separately, and they also tested the model on our same disjoint image set (PH2 [19]), achieving accuracy of 76%. This study supports evidence from previous work (see Table 4) that DL classification approaches outperform ML ones in benign vs. malignant dermoscopic skin lesion image classification. Our ML classifier performs better than the one by Bechelli and Delhomelle [9], even on a disjoint test set, but it is still not satisfactory compared with the DL approach. A possible explanation for this might be that the segmentation processing step affects the subsequent feature extraction process and it might decrease the overall accuracy of classification. Embedding segmentation in the method allows the DL model not to increase the error rate of the segmentation process.

Although the DL-based classification approach appears to have better performance than the ML-based ones in our work and also in previous studies in the literature, the better explainability and interpretability of ML models must be taken into consideration to fully evaluate and compare the two approaches. In future studies, we will work to implement explainability and interpretability techniques in the proposed DL model in order to overcome this critical issue. The better explainability and interpretability of traditional ML models could aid in gaining scientific and medical insights into the inner workings of what is still regarded as a "black box" in critical real-world applications, such as clinical ones, where it is crucial to understand and explain AI model behavior and possible related risks.

Despite this open issue relating to explainability and interpretability, both ML and DL approaches for classifying skin lesions appear to be sufficiently reliable and robust to allow for large-scale prospective clinical trials in order to demonstrate their real effectiveness, applicability, and safety in a critical clinical scenario like early melanoma diagnosis.

## 5. Conclusions

The aim of this study was the development of an AI-based solution for skin lesion image classification through the investigation of two different classification approaches: machine learning- and deep learning-based methods. The comparison between the two approaches showed better performance of the DL method in comparison with the ML one, in spite of the lesser explainability and interpretability of the DL model compared with the ML one. To evaluate the real performance and generalization properties of AI approaches, we tested both classifiers on a disjoint dataset, and we obtained performance metrics consistent with those obtained on the validation dataset. The good classification and generalization performance of both methods supports the possibility to use an automatic classification tool to support clinicians in melanoma screening. The implemented automatic classification tools could be used to overcome different clinical issues. First, an automatic analysis of skin lesion images would enable an easier and faster screening of the population. Secondly, a standardized method for benign and malignant dermoscopic image classification would decrease diagnosis variability in dermatologists with different background experience. Moreover, the AI-based approach capability to learn a massive number of data would enable the study of new common features between benign and malignant skin lesions [32].

## Figures and Tables

**Table 1:** Summary of the three datasets used for the training and testing of the models. In DB1, the number of benign skin lesion images was 65% of the entire benign dataset, and these images were randomly chosen for each of the 10 datasets. DB2 does not provide age information, and DB3 provides neither age nor sex information.

Dataset Name	DB1	DB2	DB3
Source	ISIC 2019 [15,16,17]	[18]	PH2 [19]
No. benign lesions	9640	667	160
No. malignant lesions	9281	344	40
% females	49%	52%	-
% males	51%	48%	-
Mean age	51	-	-
Used for	Training	Training	Testing

**Table 3:** Summary of data augmentation parameters and hyperparameters of the trained CNN for classification.

	Pixel range	[10, 10]
**Data augmentation**	Scale range	[0.5, 1.5]
	Rotation	[−90°, 90°]
	Frozen layers	20
**Hyperparameters**	Learning rate	0.001
	Mini-batch size	16
**Training time**	13 h	

**Table 4:** Summary of the literature-proposed methods for benign vs. malignant skin lesion dermoscopic image classification.

	Method	Dataset	Best Classification Metrics
Present study’s DL approach	CNN based on pre-trained Inception-v3	ISIC [15,16,17] and [18] for training and validation, PH2 [19] for a disjoint test dataset	Accuracy = 85.4 ± 3.2% Specificity = 75.5 ± 7.6% Precision = 93.6 ± 1.7% Recall = 88 ± 4.8%
Present study’s ML approach	Homology segmentation + ensemble boosted tree classifier	ISIC [15,16,17] and [18] for training and validation, PH2 [19] for a disjoint test dataset	Accuracy = 73.8 ± 1.1% Specificity = 44.5 ± 4.7% Precision = 85.4 ± 1.0% Recall = 81.1 ± 1.3%
Bechelli and Delhomelle, 2022	DL approach	HAM10000 dataset [15], Kaggle dataset from ISIC archive [35]	Accuracy = 88% Precision = 93% Recall = 83% F1 = 0.88
Bechelli and Delhomelle, 2022	ML approach	Kaggle dataset from ISIC archive [35]	Accuracy = 73% Precision = 57% Recall = 79% F1 = 0.66
Kaur et al., 2022	DCNN	ISIC 2016 [36], 2017 [17], and 2020 [37]; PH2 [19] for a disjoint test dataset	Accuracy = 90.4% Precision = 90.4% Recall = 90.3% On PH2: Accuracy = 76% Precision = 67.8% Recall = 75.3%
Liu et al., 2021	Mid-level feature learning based on pre-trained CNN + SVM classifier	ISIC 2017 [17]	AUC = 92.1%
Khan et al., 2020	Neural Network Classifier	Three data subsets of ISIC, ISBI 2016 [36], and PH2 [19]	Accuracy = 98.4% Precision = 98.5% F1 = 0.98
Mahbod et al., 2018	Hybrid CNN + SVM Classifier	ISIC 2016 [36] and 2017 [17]	AUC = 91.4% Accuracy = 87.7%
Premaladha and Ravichandran,2016	Neural Network + Hybrid Adaboost SVM	992 images	Accuracy = 90%

## Data Availability

All the datasets analyzed in this study are publicly archived: https://www.fc.up.pt/addi/project.html, https://challenge.isic-archive.com/data/, https://derm.cs.sfu.ca/Welcome.html accessed on 1 november 2021.

## Abbreviations

The following abbreviations are used in this manuscript:

AI	Artificial Intelligence
ML	Machine learning
DL	Deep learning
CNN	Convolutional neural network
ISIC	International Skin Imaging Collaboration
KNN	k-Nearest Neighbors
SVM	Support Vector Machine
